# Weaning Age in Yunnan Snub‐Nosed Monkeys: Effects of Interbirth Interval, Seasonality and Sex‐Biased Maternal Investment

**DOI:** 10.1002/ece3.72436

**Published:** 2025-11-11

**Authors:** Wancai Xia, Hanlan Fei, Ali Krzton, Nan He, Jinlin Ma, Dayong Li

**Affiliations:** ^1^ Key Laboratory of Conservation Biology of Rhinopithecus roxellana (Department of Education of Sichuan Province) China West Normal University Nanchong China; ^2^ Key Laboratory of Southwest China Wildlife Resources Conservation (Ministry of Education) China West Normal University Nanchong China; ^3^ Science and Technology Department of Sichuan Province Liziping Giant Panda's Ecology and Conservation Observation and Research Station of Sichuan Province Chengdu China; ^4^ Auburn University Libraries Auburn University Auburn Alabama USA; ^5^ Yunnan Baimaxueshan National Nature Reserve Diqing China

**Keywords:** IBI, seasonality, sex‐biased investment, weaning, Yunnan snub‐nosed monkeys

## Abstract

Weaning represents a critical developmental milestone in mammals, marking both the termination of maternal nutritional investment and the onset of infant nutritional independence. The weaning age may vary significantly depending on ecological and social conditions for mothers and offspring. Using 10 years of nursing data from provisioned Yunnan snub‐nosed monkeys ( *Rhinopithecus bieti* ), our results revealed a mean weaning age of 18.16 ± 3.46 months in *R. bieti* , with variation in weaning age significantly influenced by both interbirth interval (IBI) and weaning season. Regarding differences in nursing investment for different sex offspring, we found no significant difference in weaning age between male and female offspring. However, female offspring received significantly more frequent and longer nursing durations compared to males, while male offspring experienced more frequent nursing refusal. These findings demonstrate that specific behavioural and ecological conditions—including IBI and weaning season—can substantially influence weaning patterns. Our study not only enhances understanding of life history characteristics in extant *Rhinopithecus* but also provides important insights for interpreting the evolution of unique life history strategies in primates.

## Graphical abstract

Maternal lactation and influencing factors in Yunnan snub‐nosed monkeys.

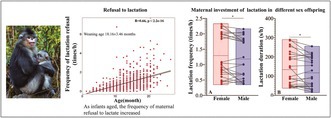

## 1 Introduction

In mammals, nursing serves as a critical mechanism through which mothers provide nutrition, immune protection, developmental modulation and gut microbiome colonisation to their offspring, supporting infant survival and conferring developmental advantages (Bădescu et al. 2022; Humphrey 2010; Pond 1977; Robbins and Robbins 2021). Consequently, weaning—defined in this study as the month of the last nipple contact, following the method of Borries et al. (2014)–represents a fundamental life history transition in mammals (Clutton‐Brock 1984; Lee 1996). Weaning age can significantly influence the fitness of both mothers and their offspring (Hayssen 1993; Kennedy 2005; Lee 1996), because it balances maternal resource allocation and offspring development. Early weaning reduces the mother's energy burden, enabling faster reproduction, but may compromise offspring growth and immunity. Delayed weaning supports offspring fitness through extended care but can deplete maternal resources and delay future reproduction. Thus, a mother's decision to invest in nursing for the current offspring or to conserve resources for future pregnancies is jointly determined by ecological factors (Fisher et al. 2002; P. C. Lee 1996; Trivers 1972a, 1972b), behavioural traits (Borries et al. 2014; Dahle and Swenson 2003; P. C. Lee 1996; Whitten 1983), nutritional status (Thompson et al. 2012) and physical growth conditions, among other factors (Eckardt et al. 2016).

Extensive research has demonstrated that weaning age in mammals is closely associated with interbirth interval (IBI) (Eckardt et al. 2016; Robbins and Robbins 2021), possibly because nursing typically suppresses ovulation and delays subsequent conception (Ziegler et al. 1990). However, recent studies highlight that the underlying mechanism driving ovulation is influenced by the energetic state of the mother (Emery Thompson 2013). This phenomenon of pregnancy before weaning has been confirmed in many species, such as *Pongo pygmaeus* (van Noordwijk et al. 2013), *Loxodonta africana* (Lee and Moss 1986), *Tursiops* sp. (Mann et al. 2000) and *Homo sapiens* (Lancaster and Alvarado 2010).

Seasonal weaning represents a pivotal adaptive strategy in mammals, particularly among seasonal breeders, shaped by evolutionary pressures to align with the cyclical availability of food resources and reproductive imperatives. This strategy reflects a fundamental trade‐off between immediate reproductive efforts and long‐term survival under resource constraints (Stearns 1992). Empirical studies indicate that weaning often coincides with periods of maximal food abundance for offspring, such as peak fruiting seasons (Di Bitetti and Janson 2000). The timing of weaning can be influenced by the seasonal availability of resources (Eckardt et al. 2016; Lee 1984), with some evidence suggesting that weaning during periods of abundance could enhance offspring survival rates (Lee 1987, 1996). This potential synchronisation between weaning and resource supply may reflect an adaptive advantage of aligning offspring development with favourable environmental conditions (Eckardt et al. 2016). Furthermore, seasonal weaning timing may be modulated by ecological and climatic stressors, as prolonged nursing during harsh winters provides essential nutritional support for offspring. Thus, seasonal weaning not only encapsulates the optimisation of maternal energy allocation and reproductive strategies but also serves as a critical adaptive mechanism to maximise offspring survival in fluctuating environments, underpinned by a robust foundation of ecological and evolutionary biological theories (Di Bitetti and Janson 2000; Eckardt et al. 2016; Lee 1984, 1987; Lee 1996).

Sex‐biased nursing investment has been widely documented (Clutton‐Brock and Guinness 1981; Hinde 2007, 2009), with several studies reporting greater maternal investment in male offspring (Bădescu et al. 2022; Eckardt et al. 2016). The generalised Trivers–Willard hypothesis posits that parents in superior condition (with greater resource‐holding potential) preferentially invest in male offspring, while those in poorer conditions favour female offspring (Trivers and Willard 1973). This aligns with the Local Resource Competition Hypothesis (Clark 1978; Hamilton 1967; Silk 1983), which predicts sex‐biased maternal investment based on dispersal patterns (Clutton‐Brock and Guinness 1981). Mothers invest more in the dispersing sex (e.g., males) to reduce local competition, while the philopatric sex (e.g., females) faces higher competition within the natal group. This differential investment reflects the trade‐off between minimising resource overlap and maximising offspring success (Clutton‐Brock and Guinness 1981; Westneat and Sargent 1996). Male‐biased nursing investment mainly involves three aspects. The first goal of male‐biased nursing investment is to improve male offspring survival rates, as males exhibit lower survival rates during stressful conditions, prompting mothers to increase their investment in males (Battles 2016; Lee and Moss 1986; Wells 2000). The second aspect is that male offspring typically demand greater maternal investment in species with pronounced sexual dimorphism due to their higher energetic requirements (Hamilton 1967; Hewison and Gaillard 1999; Trivers and Willard 1973). The third point, reproductive gain maximisation, highlights that adult males in good physical condition typically achieve higher reproductive success. However, this male‐biased strategy is high‐risk and high‐reward, involving intense competition, whereas a female‐biased strategy tends to be low‐risk and low‐reward, prioritising stability. To the extent that maternal investment influences this outcome, mothers are expected to allocate more resources to male offspring in exchange for greater reproductive gain. This strategy reflects an evolutionary adaptation to the transmission of maternal genes by investing in offspring that are likely to yield higher reproductive returns (Hewison and Gaillard 1999; Trivers and Willard 1973). However, Bădescu et al. (2022) observed greater reproductive skew towards males compared to females. Furthermore, male offspring exhibited higher variability in reproductive outcomes than their female counterparts (Bădescu et al. 2022). In general, mothers benefit from sex‐biased investment towards the offspring sex that yields higher reproductive returns—a strategy shaped by life history traits and environmental conditions. For example, in *Bos taurus* , females reproduce before males; therefore, mothers invest more in nursing for female offspring to accelerate their reproductive onset (Hinde et al. 2014).

The Yunnan snub‐nosed monkey ( *Rhinopithecus bieti* ), endemic to the Hengduan Mountains of China, is the non‐human primate with the highest altitudinal range in the world (3000–4700 m a.s.l.) (Xia et al. 2020). This endangered species displays a multilevel social system comprising one‐male units (OMUs) and all‐male units (AMU) (Xia et al. 2020), with females philopatric and males dispersing (Xia et al. 2022). As the sole caregivers, females' reproductive investment—particularly nursing strategies—directly affects offspring survival and population viability (Xia et al. 2022). To investigate weaning patterns in *R. bieti* , we analysed a 10‐year behavioural data set (2013–2022) from a provisioned population in Xiangguqing, Baimaxueshan National Nature Reserve. Our study addressed four objectives: (1) to determine the empirical weaning age of this species, (2) to investigate the correlation between weaning and IBI, (3) to assess whether *R. bieti* weans during seasons of food abundance, (4) to analyse the sex‐based disparities in nursing investment in *R. bieti* .

## 2 Materials and Methods

### 2.1 Study Site and Study Subjects

**FIGURE 1:**
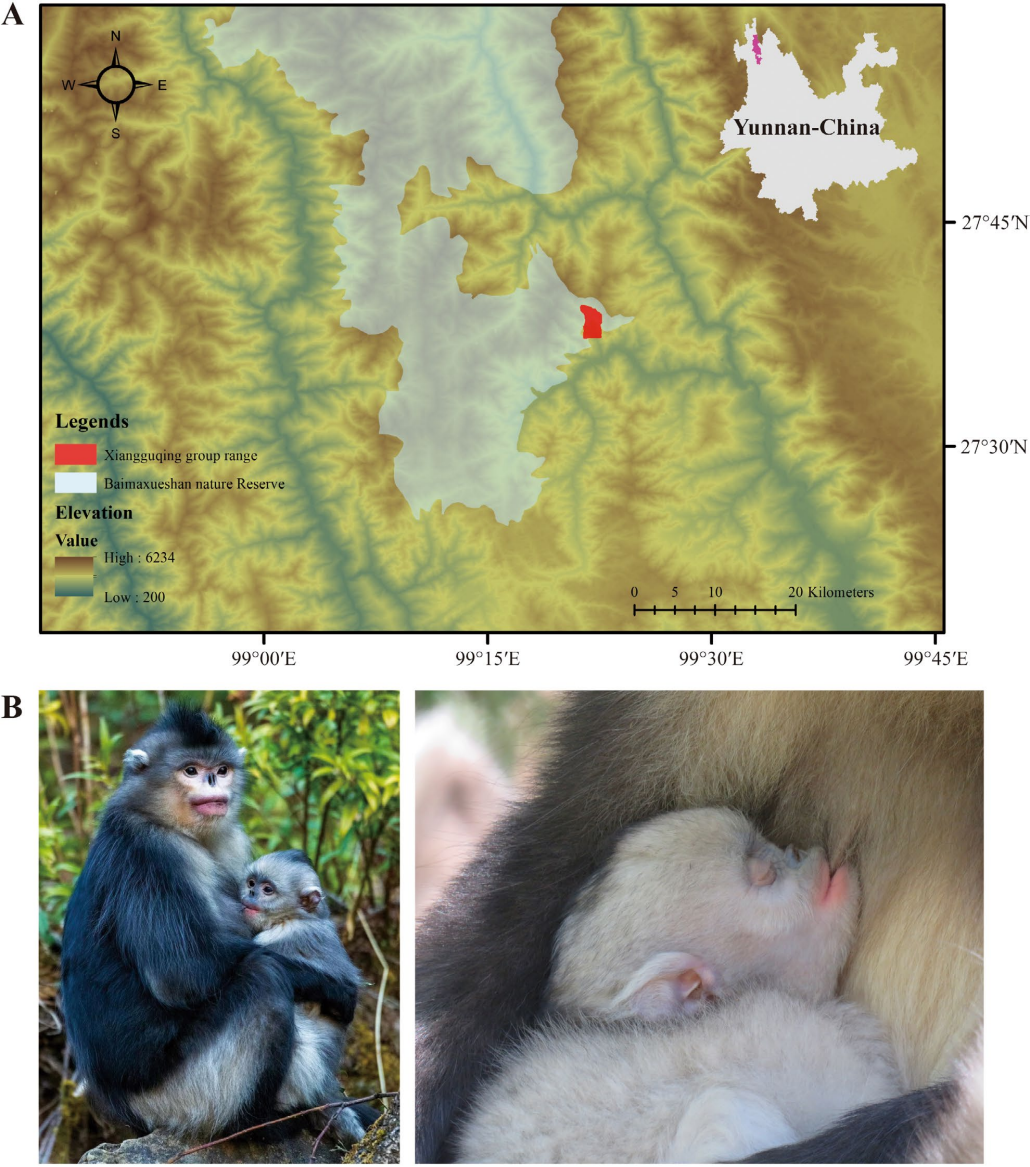
Study area and focal species. (A) Geographic location of the Xiangguqing study site in Baimaxueshan National Nature Reserve, Yunnan, China. (B) Lactating female *R.bieti* with infant. Photograph credit: Wancai Xia.

This study was conducted in Xiangguqing (99° 22′ E, 27° 37′ N), located within Baimaxueshan National Nature Reserve, northwestern Yunnan, China (Figure 1A). The topography of the area features a V‐shaped, covering approximately 90 km^2^ (Li et al. 2014; Xia et al. 2016). The region contains mixed coniferous and deciduous broadleaf forest, subalpine fir forest, montane sclerophyllous oak forest, subtropical evergreen broadleaf forest and pine forest, as well as other habitat types (Xia et al. 2016). The study site lies on the southeast extension of the Qinghai–Tibet Plateau, characterised by a plateau climate (cold temperate zone). The monsoon climate dominates, with distinct dry and wet seasons, limited annual temperature variation, and significant diurnal temperature fluctuations (Xia et al. 2016). Following plant phenology characteristics, the seasons are defined as: spring (March–May), summer (June–August), autumn (September–November) and winter (December–February) (Li et al. 2014). The diet of Yunnan snub‐nosed monkeys varies seasonally, with tender leaves as the main food in spring, bamboo shoots and mature leaves in summer, fruits and seeds in autumn, and lichens (*Usnea* spp.) in winter. Food diversity is highest in summer and lowest in winter (Li et al. 2011).

The focal monkey group, known as the stable provisioned group, separated from a larger local natural group in May 2008 (Ren et al. 2012). During the study period, reserve staff provided food to the monkey group at 09:00 and 17:00 daily, consisting of: 10 kg lichens, 3 kg carrots, 3 kg corn and 1.5 kg pumpkin seeds, supplemented with bamboo shoots in June and July (Xia et al. 2016). This provisioning strategy (total~17.5 kg/day for the group) represented < 30% of estimated daily intake. These *R. bieti* individuals become habituated to researcher presence, enabling reliable identification of each individual based on distinctive physical characteristics, including body size, pelage patterns, scars and facial features (Xia et al. 2020). Since 2010, long‐term behavioural monitoring has included reproduction, dispersal patterns, dominance hierarchies and social grooming, leading to comprehensive life history records for each individual.

From 2013 to 2022, we observed 22 one male units (OMUs) and one all male unit (AMU), with group sizes ranging from 42 to 80 individuals. During this period, 91 infants were born, including seven that were stillborn, six neonatal deaths (0–6 months) and three cases of mother–offspring dispersal (Table S1). An additional seven infants had not yet been weaned by the end of 2022, resulting in a final 68 infants with complete developmental records from birth to weaning.

### 2.2 Behavioural Observations

From 2013 to 2022, seven trained postgraduate students successively conducted the collection of behavioural data. The provisioned group was fully habituated to researcher presence, allowing close‐range observations (typically 5–15 m) with 8 × 42 binoculars (Olympus) used when necessary. We employed all‐occurrence sampling (Altmann 1974) to record nursing behaviours, rotating observations among OMUs daily from 09:00 to 17:00 (weather and terrain permitting). For this study, we defined nursing operationally as the infant maintaining contact with the mother's nipple in its mouth (Figure 1B). While some studies require visible swallowing as a criterion, we found this unfeasible under field conditions. Each suckling event was timed using digital stopwatches, with durations recorded to the nearest second. We employed the criteria established by Borries et al. (2014) to define weaning, specifically using the age in months at the last observed nipple contact. In this study, weaning was confirmed when no nipple contact was observed for a continuous period of 1 month.

During each observation session, we systematically documented: participant identification (infant's name, sex, mother's name), nursing duration (from nipple contact to release), nursing refusal (mother actively preventing nipple access) (Li et al. 2014). Demographic parameters were obtained from our long‐term database: date of birth of the infant and IBI (Xia et al. 2019, 2022).

### 2.3 Infants Age and IBI

The infant's age and interbirth interval (IBI) were calculated based on our long‐term observation database (Xia et al. 2019), moreover, we were able to precisely determine each reproductive event to the day (with a potential margin of error of 1–2 days). The IBI (in months) measures the time (in months) between a female's two most recent reproductive events (Table S2), including stillbirths and premature death, though the data is often considered artificially shortened (Borries et al. 2001).

### 2.4 Nursing Data Standardisation Processing

Due to challenges posed by inclement weather conditions, loss of animal tracking and other logistical constraints, systematic uniform sampling could not be consistently maintained throughout the study period. To address these limitations and ensure robust data analysis, we implemented a monthly aggregation approach for the collected nursing behaviour data. Specifically, we calculated three key monthly metrics: (1) nursing frequency (number of nursing events per month), (2) nursing duration (average time spent per nursing session), (3) nursing refusal rate (frequency of nursing attempts that were rejected). These monthly aggregated metrics were subsequently integrated into our comprehensive statistical analysis framework to ensure meaningful interpretation of the behavioural patterns while accounting for sampling variability.

$$F_{\text{nursing}}=N_{\text{nursing}}/T.$$

*F*_nursing_, Frequency of nursing; *N*_nursing_, number of nursing bouts; *T*, observation time.

$$D_{\text{nursing}}=D_{\text{nipple contact}}/T.$$

*D*_nursing_, Duration of nursing; *D*_nipple contact_, duration of nipple contact of offspring; *T*, observation time.

$$F_{\text{refusal}}=N_{\text{refusal}}/T.$$

*F*_refusal_, Frequency of refusal to nursing; *N*_refusal_, number of refusals; *T*, observation time.

### 2.5 Data Analysis

The average weaning age (Mean ± SD) was the mean age (in months) of all observed individuals who have been weaned. To examine the differences in weaning age between male and female offspring, an independent samples t‐test was conducted. For the analysis of seasonal variations in weaning age, we first assessed the homogeneity of variances using Levene's test. Given the heterogeneity of variances across seasons in the weaning age data, the Kruskal–Wallis test, a non‐parametric alternative, was employed to evaluate the overall differences. Additionally, Dunn's test, a post hoc analysis, was performed to identify specific pairwise differences between seasons. The Pearson correlation test was utilised to explore the relationships between weaning age and interbirth interval (IBI), as well as between the frequency of nursing refusal and the age of the offspring. Furthermore, a paired sample t‐test was performed to assess differences in nursing frequency, nursing duration, nursing refusal frequency between male and female offspring of the same age, utilising the mean values for each age group in the comparative analysis.

## 3 Results

### 3.1 Weaning Age and Interbirth Interval (IBI)

**FIGURE 2:**
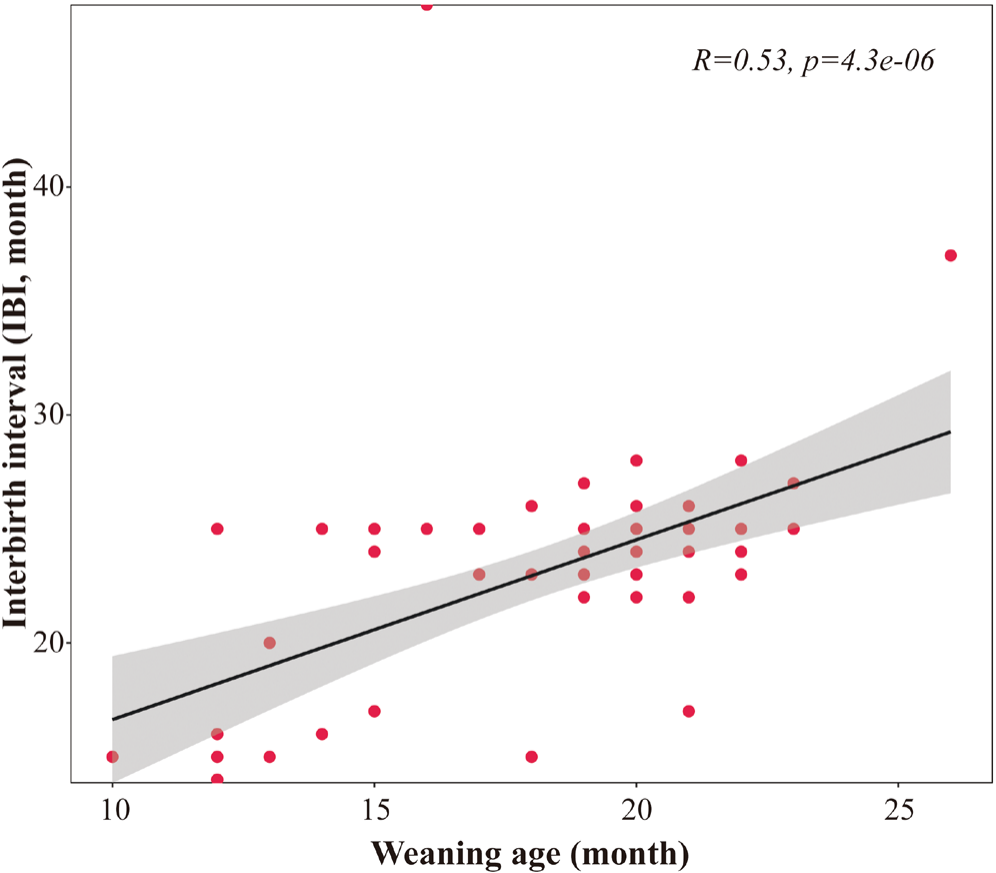
The positive correlation between weaning age and IBI.

From 2013 to 2022, during a total of 5808.18 h of observation, we recorded 7171 nursing events, totalling 212.79 h of nursing and 2657 instances of nursing refusal. The mean weaning age was 18.16 ± 3.46 months (range: 10–26 months, *n* = 68). The Pearson correlation test indicates that IBI (range: 14–48 months) is significantly positively correlated with the weaning age (*R* = 0.525167, *p* < *0.001, n* = 68, Figure 2).

### 3.2 Seasonal Variations in Weaning

**FIGURE 3:**
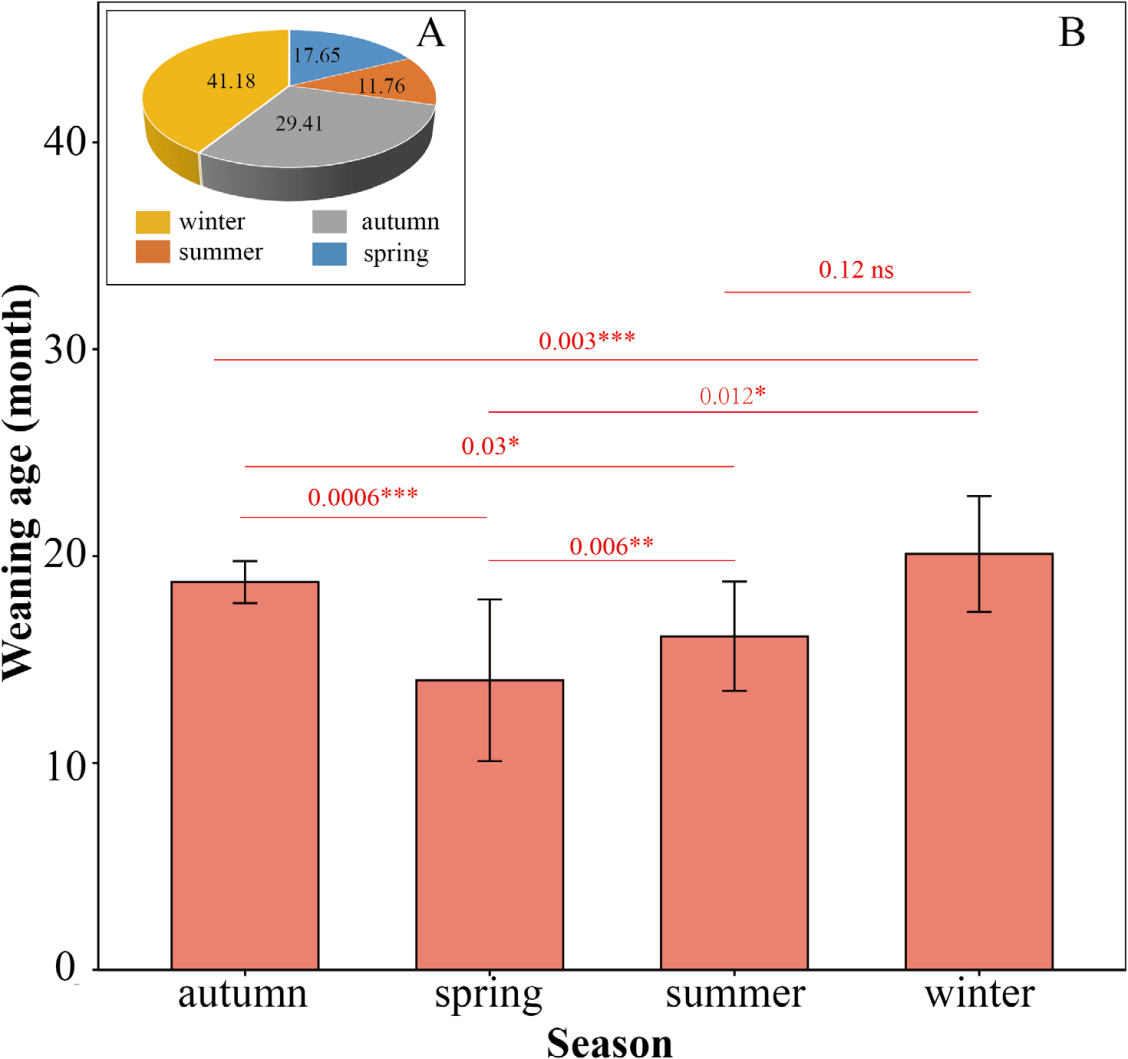
Seasonal variations in weaning age. (A) seasonal distribution of weaning, (B) weaning ages in different seasons. The figure shows the significance after Bonferroni *p*‐value correction, where * indicates significance, ** indicates high significance, *** indicates extremely significant and ns indicates no significance.

The analysis indicates that the weaning season of the research monkey group does not seem to coincide with the season of high food abundance. The Chi‐squared test found that the distribution of weaning events across seasons is not uniform (*χ* ^2^ = 12.941, df = 3, *p* = 0.005). Weaning mainly occurred during the winter when food is scarce, and the proportion of weaning in other seasons is shown in Figure 3A. The weaning ages exhibited significant seasonal variations (Kruskal–Wallis test, *H* = 25.73, df = 3, *p < 0.001*). The multiple comparison results of the Dunn's test analysis showed that the age of spring‐weaned individuals (Mean ± SD = 14.00 ± 3.91, *n* = 12) was significantly younger than that of summer (Mean ± SD = 16.13 ± 2.64, *n* = 8, *p* = 0.006), autumn (Mean ± SD = 18.75 ± 1.02, *n* = 20, *p* < 0.001) and winter (Mean ± SD = 20.11 ± 2.79, *n* = 28, *p* < 0.001). The weaning age in winter was significantly higher than that in other seasons. There was no significant difference in the weaning age between summer and autumn (Figure 3B).

### 3.3 The Sex‐Biased Maternal Investment in Nursing

**FIGURE 4:**
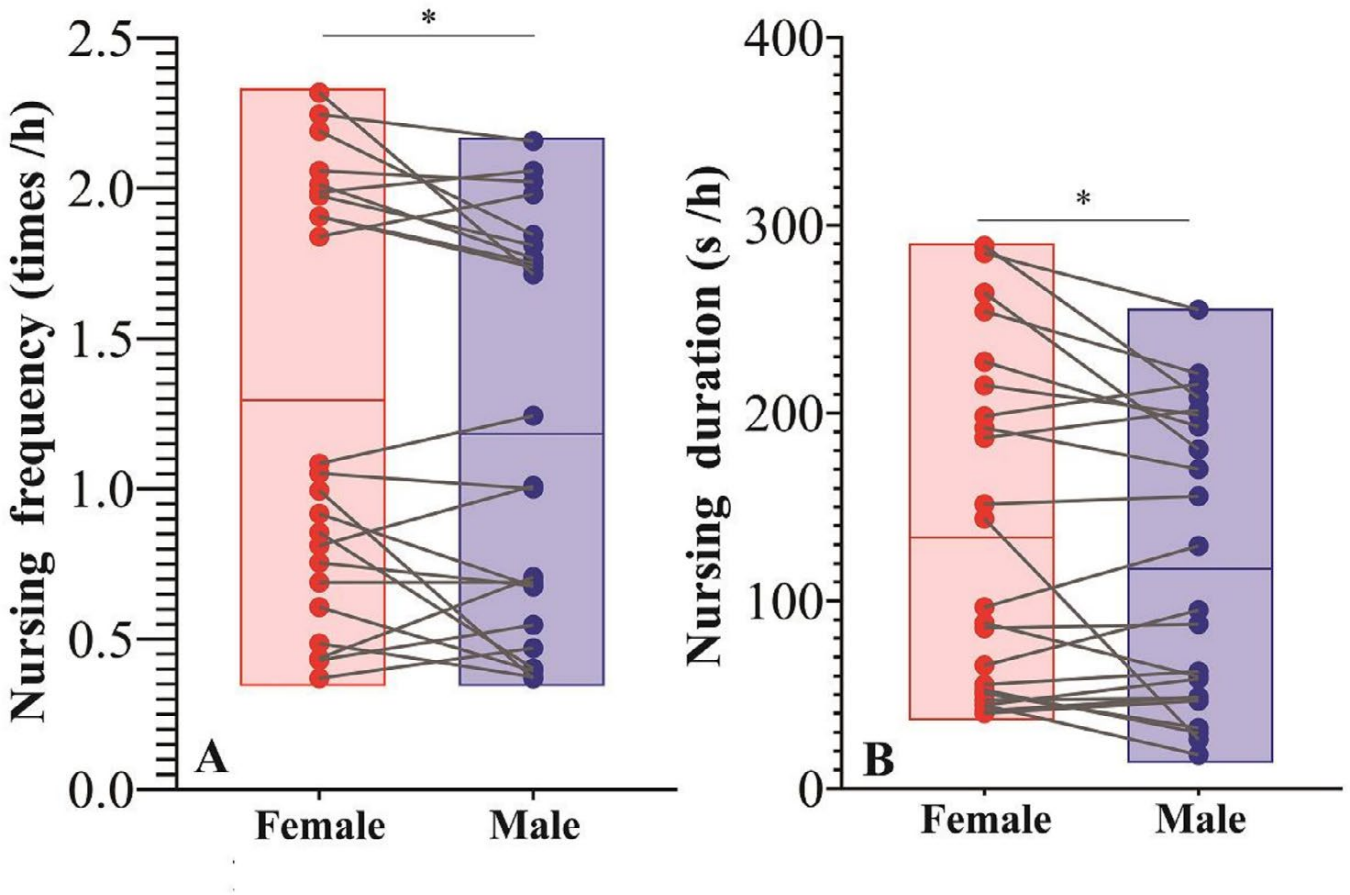
Nursing frequency and duration by sex. (A) differences in nursing frequency of different sex offspring. (B) differences in nursing duration of different sex offspring. *Represents *p* < 0.05.

In the analysis of offspring sex differences, there was no significant difference in the mean weaning age of males (Mean ± SE = 18.78 ± 3.20 months, *n* = 36) compared to females (Mean ± SE = 17.47 ± 3.65 months, *n* = 32, *p* = 0.751). However, there were differences in nursing frequency and duration by sex. Since the maximum observed weaning age for male infants was 23 months, we performed a paired sample t‐test to compare nursing frequency and duration between male and female offspring of the same age (1–23 months). The results showed that female offspring had a significantly higher nursing frequency (Mean ± SE = 1.297 ± 0.145) than male offspring (Mean ± SE = 1.192 ± 0.138, *t* = 2.081, *p* < 0.05, Figure 4A). Similarly, the paired sample *t*‐test for nursing duration showed that female offspring nursed significantly longer (Mean ± SE = 122.46 ± 17.68) compared to male offspring (Mean ± SE = 105.57 ± 16.24, *p* = 0.021, Figure 4B).

**FIGURE 5:**
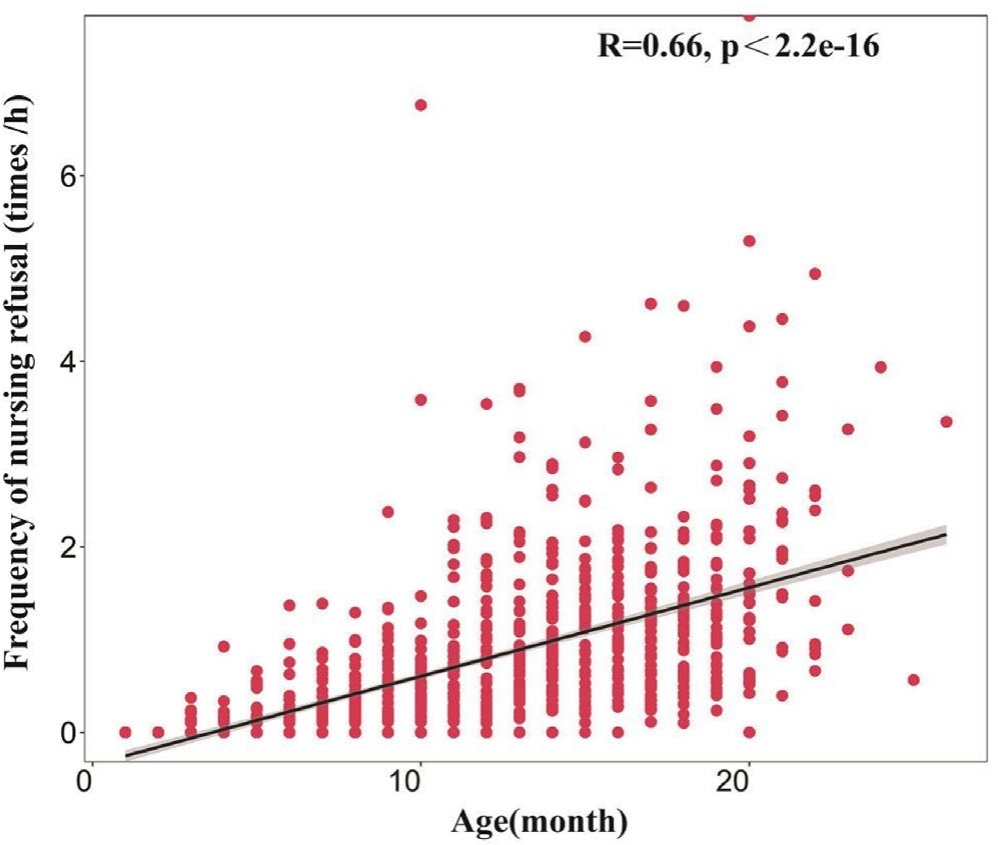
Relationship between offspring age and maternal refusal to lactate. The abscissa represents the age of the offspring in months, and the ordinate represents the frequency of nursing refusal.

As infants aged, the frequency of maternal refusal to lactate increased significantly (*R* = 0.66, *p* < 0.01, Figure 5). We conducted a paired sample *t*‐test (1–23 months) on the frequency of refusal of different‐sex offspring of the same age. The results showed that female offspring were refused nursing less frequently (Mean ± SE = 0.692 ± 0.122) than male offspring (Mean ± SE = 0.916 ± 0.165, *t* = 3.436, *p* = 0.002).

## 4 Discussion

Nursing provides essential nutritional and immunological benefits to infants (Humphrey 2010; Pond 1977), establishing weaning as a pivotal life history event in mammals (Clutton‐Brock 1984; P. C. Lee 1996). Our findings suggest that weaning age in provisioned *R. bieti* is associated with certain aspects of reproductive strategies, as the interbirth interval (IBI) increases with later weaning. This pattern aligns with observations in Phayre's leaf monkeys (Borries et al. 2014) and Virunga mountain gorillas (Eckardt et al. 2016). Nevertheless, the observed positive relationship between extended nursing periods and longer IBI is more likely related to maternal energy status than to the hypothesis that nursing inhibits ovulation, as prolonged nursing may delay subsequent reproduction due to increased energetic demands (Ziegler et al. 1990). On average, *R. bieti* exhibit an IBI of approximately 2 years (Xia et al. 2019), which is slightly shorter than the combined duration of the gestation period (6.8 months) (He et al. 2001) and the nursing period (18.16 ± 3.46 months). These findings reveal that *R.bieti* can conceive without weaning the previous infant, a phenomenon also documented in *Pongo pygmaeus* (van Noordwijk et al. 2013), *Loxodonta africana* (Lee and Moss 1986), *Tursiops* sp. (Mann et al. 2000) and *Homo sapiens* (Lancaster and Alvarado 2010).

However, recent research revealed that the mechanisms driving ovulation are not solely dependent on nursing frequency but are closely linked to the maternal energy status (Emery Thompson 2013). When maternal energy requirements are sufficiently met, pregnancy can occur during nursing, enabling a shortened interbirth interval (IBI) as the mother can conceive while still nursing the previous offspring. This phenomenon has been widely documented in multiple species, such as *Pongo pygmaeus* (van Noordwijk et al. 2013), *Loxodonta africana* (Lee and Moss 1986), *Tursiops* sp. (Mann et al. 2000) and *Homo sapiens* (Lancaster and Alvarado 2010). The *R. bieti* typically exhibits an average IBI of 2 years (Xia et al. 2019). However, during the study period, eight cases of IBI lasting only 1 year—involving pregnancies during nursing—were recorded (Table S1). In these cases, maternal monkeys were forced to wean their previous offspring prematurely to nurse the newborn and ensure its survival. This adaptive strategy not only reduced the weaning age but also shortened the IBI. In female *R. bieti* with an interbirth interval (IBI) exceeding 2 years, the prolonged nursing period can be explained by two key factors. First, nursing is extended into the second year of the infant's life only if the mother fails to conceive within a limited reproductive period (Gomendio 1989; Johnson et al. 1993). Second, infants may continue to suckle the nipple even after the mother has ceased milk production, with the act primarily providing emotional or psychological comfort rather than nutritional sustenance.

Seasonal food availability significantly influences the timing of weaning (Eckardt et al. 2016; Lee 1984). While studies suggest that weaning during periods of abundant food resources enhances offspring survival (Lee 1987, 1996), our findings reveal a unique pattern in *R.bieti*, where weaning predominantly occurs during the food‐scarce winter, diverging from the peak food availability period. This deviation may be attributed to the pronounced seasonal fluctuations in food supply within their habitat. Spring, summer and autumn are characterised by abundant food resources, such as buds, young leaves, bamboo shoots and fruits, while winter is marked by severe food scarcity, during which individuals rely on low‐energy foods like bark and lichens (Li et al. 2014). Our results demonstrate pronounced seasonal variations in weaning ages, with winter‐weaned offspring showing the longest nursing period compared to other seasons. In contrast, spring exhibited the shortest nursing period, with weaning occurring earlier, while summer and autumn displayed intermediate weaning ages. These patterns align with the broader understanding that weaning age is closely linked to habitat food quality (Lycett et al. 1998). The strategic reduction of nursing duration during spring, summer and autumn enhances offspring survival while conserving maternal energy for future reproduction. This mirrors observations in *Papio cynocephalus ursinus* (Lycett et al. 1998) and *Arctocephalus galapagoensis* (Trillmich 1986), where abundant food resources enable earlier weaning by supporting independent foraging (Trillmich 1986).

During winter, the extension of the nursing period acts as a vital nutritional safeguard, ensuring infants are protected from starvation during this time of environmental scarcity (Eckardt et al. 2016; P. Lee 1984). However, the provision of artificially supplemented high‐nutrient foods may have alleviated energy constraints during the weaning period, potentially enabling *R. bieti* to successfully complete weaning even during winter. This suggests that human intervention may disrupt natural weaning dynamics, even under conditions of seasonal resource scarcity. Further research incorporating direct metrics of food availability is needed to confirm this relationship and explore the underlying mechanisms.

Seasonal weaning, as an adaptive strategy in mammals, is traditionally shaped by evolutionary pressures to align with cyclical resource availability and reproductive demands (Stearns 1992). While empirical studies have consistently demonstrated that weaning typically coincides with periods of maximal food abundance (Di Bitetti and Janson 2000; Eckardt et al. 2016; Lee 1984, 1987; Lee 1996), our findings reveal a potential deviation in *R.bieti* due to the provision of artificially supplemented high‐nutrient foods. Additionally, our results highlight the role of ecological and climatic stressors, such as prolonged nursing during harsh winters, in modulating weaning strategies. These insights contribute to the broader understanding of how maternal energy allocation and offspring survival are optimised in fluctuating environments, while also emphasising the potential impacts of anthropogenic factors on natural behaviours.

Sex‐biased maternal investment (Clutton‐Brock and Guinness 1981) represents an adaptive strategy that maximises maternal and offspring fitness (Westneat and Sargent 1996). In species with pronounced sexual dimorphism, theory predicts differential maternal investment favouring male offspring (Hamilton 1967; Hewison and Gaillard 1999; Trivers and Willard 1973), a pattern empirically supported in various mammals (Hewison and Gaillard 1999). In contrast, our results suggest that although weaning age does not differ significantly by sex, mothers invest more in their female offspring, as evidenced by nursing frequency, duration and the frequency of nursing refusal. While our study did not compare the nutritional content of milk, we found no difference in nursing frequency or duration between male and female offspring under 1 year of age (Table S2). Sex differences in nursing investment become apparent after 1 year, once offspring have begun feeding independently (attempts begin around 6 months of age) and male offspring have survived the ‘fragile male’ period (Battles 2016).

Owing to the polygynous nature of *R. bieti* , males disperse much farther than females at sexual maturity, resulting in a loose matrilineal social system (Xia et al. 2022). Increased investment in female offspring facilitates the establishment of a matrilineal alliance. While the literature generally supports the idea that mothers invest more in male offspring in species with pronounced sexual dimorphism (Hamilton 1967; Hewison and Gaillard 1999; Trivers and Willard 1973), *R.bieti* exhibits male dispersal and female philopatry in *R.bieti* (Xia et al. 2022). In this context, mothers tend to prioritise investment in philopatric females rather than males, who face greater uncertainty when seeking new social groups to achieve reproductive success. Second, in polygamous species, the variance in male reproductive success is typically greater than that of females (Bădescu et al. 2022). Since male *R. bieti* offspring may or may not become OMU leaders after sexual maturity, and the length of tenure for OMU leaders varies (Xia et al. 2021), it is plausible that male reproductive skew in this species could be high. In contrast, female reproductive success in *R. bieti* is relatively even, as nearly every female gives birth to one infant every 2 years (Xia et al. 2019). Although previous studies have demonstrated that females can benefit from increased investment in their male offspring (Clutton‐Brock 1984), female *R. bieti* appear to adopt a distinct, conservative nursing strategy characterised by female‐biased investment. This strategic approach may serve to enhance nursing efficiency, ensuring stable reproductive returns and fostering future social cooperation, while simultaneously maximising offspring survival rates.

Our decade‐long observation of nursing behaviours in a wild provisioned group of Yunnan snub‐nosed monkeys has revealed the factors influencing weaning age and their interaction with maternal investment and reproductive strategies. The study found that the average weaning age was 18.16 months and weaning age showed a positive correlation with the IBI. Weaning timing exhibited pronounced seasonal variations, with most weaning occurring during the food‐scarce winter, likely due to artificial supplemental feeding alleviating energy constraints. Additionally, maternal investment displayed sex‐biased patterns, with female offspring receiving more nursing attention than males, potentially reflecting the species' social structure and female philopatry. These findings highlight the adaptive strategies of *R. bieti* in balancing reproductive success, offspring survival and environmental challenges, providing important insights into the evolutionary pressures shaping primate life history traits.

## Author Contributions

**Wancai Xia:** conceptualization (equal), data curation (equal), formal analysis (equal), funding acquisition (equal), investigation (equal), methodology (equal), project administration (equal), resources (equal), software (equal), supervision (equal), validation (equal), visualization (equal), writing – original draft (equal), writing – review and editing (equal). **Hanlan Fei:** conceptualization (equal), software (equal), visualization (equal), writing – review and editing (equal). **Ali Krzton:** methodology (equal), writing – review and editing (equal). **Nan He:** data curation (equal), investigation (equal), methodology (equal), resources (equal), software (equal), visualization (equal). **Jinlin Ma:** data curation (equal), investigation (equal), resources (equal), software (equal), writing – review and editing (equal). **Dayong Li:** conceptualization (equal), investigation (equal), methodology (equal), resources (equal), software (equal), supervision (equal), writing – original draft (equal), writing – review and editing (equal).

## Ethics Statement

Our study constitutes non‐invasive behavioural research, which has been approved by the Research Ethics Committee of China West Normal University (Approval No. CWNU20100001).

## Conflicts of Interest

The authors declare no conflicts of interest.

## Supporting information


**Table S1:** Information on the Study Subjects.

**Table S2:** Information on Weaning and Reproduction of Newborns.

**Data S1:** ece372436‐sup‐0003‐DataS1.xlsx.

**Data S2:** ece372436‐sup‐0004‐DataS2.xlsx.

## Data Availability

The data that supports the findings of this study are available in the Supporting Information of this article.
