# Supplementary material for: Weaning Age in Yunnan Snub‐Nosed Monkeys: Effects of Interbirth Interval, Seasonality and Sex‐Biased Maternal Investment
Source: Ecol Evol. 2025 Nov 11;15(11):e72436. doi: 10.1002/ece3.72436 (PMC12604898; doi:10.1002/ece3.72436)
Supplement: Supplementary file 1 — Table S1: Information on the Study Subjects. [file ECE3-15-e72436-s001.docx]

**Table S1 Information on the study subjects**

|  | ID | Date of birth | Sex | ID of mother | OMU | Note |
| --- | --- | --- | --- | --- | --- | --- |
| 1 | 13-1 | 2013.3 | female | ximao | DGZ |  |
| 2 | i1 | 2014.2 | female | jiyin | DB | Died three months after birth |
| 3 | 14-1 | 2014.2 | female | baibi | HL |  |
| 4 | 14-2 | 2014.2 | male | endeng | HL |  |
| 5 | 14-3 | 2014.3 | female | baimei | HL |  |
| 6 | 14-4 | 2014.3 | female | jihao | DB |  |
| 7 | 14-5 | 2014.3 | female | baiyuding | DB |  |
| 8 | 14-6 | 2014.3 | female | baisi | LHG |  |
| 9 | 14-7 | 2014.4 | male | pianlian | DS |  |
| 10 | 15-1 | 2015.3 | male | douyb | DGZ |  |
| 11 | 15-2 | 2015.3 | male | ximao | DGZ |  |
| 12 | 16-1 | 2016.2 | female | xiaohei | HD |  |
| 13 | 16-2 | 2016.2 | female | lingjia | HL |  |
| 14 | 16-3 | 2016.2 | female | baimei | HL |  |
| 15 | 16-4 | 2016.2 | female | baibi | HL | Died six months after birth |
| 16 | 16-5 | 2016.3 | female | baiyuding | LB |  |
| 17 | 16-6 | 2016.3 | female | pianlian | DS |  |
| 18 | 16-7 | 2016.3 | female | maolian | HD |  |
| 19 | 16-8 | 2016.4 | male | lingyi | HD |  |
| 20 | i2 | 2016.4 |  | baomu | XS | Died five months after birth |
| 21 | 16-9 | 2016.4 | male | endeng | HL |  |
| 22 | 16-10 | 2016.5 | male | douyb | DGZ |  |
| 23 | i3 | 2016.5 |  | lingxin | HD | Stillbirth |
| 24 | 16-11 | 2016.5 | male | baisi | LHG |  |
| 25 | 17-1 | 2017.2 | male | lingxin | HD |  |
| 26 | 17-2 | 2017.3 | female | jihao | DB |  |
| 27 | 17-3 | 2017.3 | male | chunfen | HD |  |
| 28 | 17-4 | 2017.3 | female | lingkui | XS |  |
| 29 | 17-5 | 2017.3 | male | eryi | XS |  |
| 30 | 17-6 | 2017.4 | male | baomu | XS |  |
| 31 | 17-7 | 2017.4 | male | xiaohei | HD |  |
| 32 | 17-8 | 2017.4 | male | ximao | DGZ |  |
| 33 | 17-9 | 2017.5 | male | maolian | HD |  |
| 34 | 18-1 | 2018.2 | female | endeng | HL |  |
| 35 | 18-2 | 2018.2 | female | douyb | DGZ |  |
| 36 | 18-3 | 2018.3 | male | baiyuding | LB | Move out with mother before weaning |
| 37 | 18-4 | 2018.3 | male | lingjia | SG |  |
| 38 | i4 | 2018.3 | male | pianlian | DS | Stillbirth |
| 39 | 18-5 | 2018.3 | male | lingyi | HD |  |
| 40 | 18-6 | 2018.4 | male | erding | HD |  |
| 41 | 18-7 | 2018.4 | female | baisi | LHG | Move out with mother before weaning |
| 42 | 18-8 | 2018.4 | female | lingkui | XS |  |
| 43 | 18-9 | 2018.5 | male | baimei | HL |  |
| 44 | 18-10 | 2018.5 | female | sanniu | ML |  |
| 45 | 18-11 | 2018.6 | female | erbing | XS |  |
| 46 | 19-1 | 2019.2 | male | jihao | DB |  |
| 47 | 19-2 | 2019.2 | male | maolian | HD |  |
| 48 | 19-3 | 2019.2 | male | ximao | DGZ |  |
| 49 | 19-4 | 2019.3 | female | baomu | XS |  |
| 50 | 19-5 | 2019.3 | female | eryi | XS |  |
| 51 | i5 | 2019.3 | male | chunfen | HD | Stillbirth |
| 52 | 19-6 | 2019.3 | male | siji | SG |  |
| 53 | 19-7 | 2019.4 | female | sijia | ML |  |
| 54 | 19-8 | 2019.5 | male | douyb | DGZ |  |
| 55 | 19-9 | 2019.5 | male | lingxin | HD |  |
| 56 | 19-10 | 2019.5 | male | xiaohei | HD |  |
| 57 | 20-1 | 2020.1 | male | lingyi | HD |  |
| 58 | 20-2 | 2020.2 | male | endeng | WG |  |
| 59 | 20-3 | 2020.2 | male | chunfen | EW |  |
| 60 | 20-4 | 2020.3 | female | lingjia | SG |  |
| 61 | 20-5 | 2020.3 | male | erbing | XS | Move out with mother before weaning |
| 62 | 20-6 | 2020.3 | female | sibing | ML |  |
| 63 | 20-7 | 2020.3 | female | siding | BL |  |
| 64 | 20-8 | 2020.3 | female | sanniu | ML |  |
| 65 | 20-9 | 2020.4 |  | baomu | XS | Stillbirth |
| 66 | 20-10 | 2020.5 |  | lingkui | XS | Stillbirth |
| 67 | 20-11 | 2020.6 | female | maolian | EW |  |
| 68 | 21-1 | 2021.2 | female | liuyi | SG |  |
| 69 | 21-2 | 2021.2 |  | jihao | DB | Died two months after birth |
| 70 | 21-3 | 2021.2 | female | ximao | DGZ |  |
| 71 | 21-4 | 2021.3 | male | sijia | ML |  |
| 72 | 21-5 | 2021.3 | male | xiaohei | HD |  |
| 73 | 21-6 | 2021.3 | male | lingxin | HD |  |
| 74 | 21-7 | 2021.3 | female | douyb | DGZ |  |
| 75 | 21-8 | 2021.7 |  | liujia | HBZ | Stillbirth |
| 76 | 21-9 | 2021.4 | male | siji | SG |  |
| 77 | 21-10 | 2021.4 | female | lingyi | HD |  |
| 78 | 21-11 | 2021.5 | male | sanniu | ML |  |
| 79 | 21-12 | 2021.7 | female | lingjia | SG |  |
| 80 | 21-13 | 2021.8 | male | xinjiu | EJ |  |
| 81 | 22-1 | 2022.2 | male | siding | QG |  |
| 82 | 22-2 | 2022.2 | female | liubing | EJ | died two months after birth |
| 83 | 22-3 | 2022.2 | male | sibing | ML | Not weaned |
| 84 | 22-4 | 2022.3 | male | maolian | QG | Not weaned |
| 85 | 22-5 | 2022.3 | male | chunfen | HBZ | Not weaned |
| 86 | 22-6 | 2022.3 | male | baimei | EJ | Not weaned |
| 87 | 22-7 | 2022.4 | female | qiyi | HBZ | Not weaned |
| 88 | 22-8 | 2022.4 | female | qiqi | EJ | Not weaned |
| 89 | 22-9 | 2022.5 | male | lingxin | HD | Not weaned |
| 90 | 22-10 | 2022.5 |  | xiaohei | HD | Stillbirth |
| 91 | 22-11 | 2022.2 | male | sijia | ML | Died one months after birth |
